# NEO212: sub-cytotoxic doses capable of inhibiting glioma stem cell invasion

**DOI:** 10.18632/oncoscience.429

**Published:** 2018-06-27

**Authors:** Nagore I. Marín-Ramos, Florence M. Hofman, Thomas C. Chen

**Affiliations:** Department of Neurosurgery, Keck School of Medicine, University of Southern California, Los Angeles, CA 90033, USA

**Keywords:** glioblastoma, glioma stem cells (GSC), temozolomide (TMZ), NEO212, invasion

Glioblastoma multiforme (GBM) is a malignant brain tumor characterized by its extensive vascularity, aggressiveness and invasiveness, where cell migration plays an important role in tumor progression. Its poor prognosis is associated with high recurrence, and resistance to the current standard of care chemotherapy, temozolomide (TMZ). Despite great progress made in surgery and therapies, there has been little improvement to patient outcome over the past decade [1]. Once the tumor recurs, there are few treatment options available to patients. Thus, chemotherapeutic agents with greater efficacy than TMZ are badly needed.

GBM cells often acquire resistance to TMZ through mutations in the DNA repair mechanisms such as the base excision repair (BER), poly(ADP-ribose) polymerase (PARP), mismatch repair (MMR), and especially, through the overexpression of the repair enzyme O^6^-methyl-guanine-DNA methyltransferase (MGMT) [2]. One of the main causes for tumor recurrence is a small sub-population of TMZ-resistant glioma stem cells (GSC), with capacity for self-renewal and *in vivo* tumor initiation [3, 4]. NEO212, a conjugate of TMZ and perillyl alcohol (POH), has proven to be active against TMZ-resistant glioma cells, including GSC [5, 6]. This compound exerts a more potent DNA alkylating activity than TMZ that causes cytotoxicity during the first hours of treatment [7], and is able to overcome the mechanisms of TMZ-resistance [5, 6].

We have recently reported that NEO212 also decreases migration and invasion of several patient-derived GSC to a greater extent than TMZ and/or POH, through a mechanism independent of its DNA alkylating effects [2]. TMZ loses its cytotoxicity after 2 h when incubated in cell-free medium at 37°C, while NEO212 loses it after 24 h [8]. However, the effects of NEO212 on GSC migration are maintained after this 24 h-incubation, which suggests that one or more of the breakdown products of NEO212 could be responsible for the blockade of GSC migration. This reduction in migration and invasion is likely associated with the decreased activation of the FAK/Src signaling pathway, as shown by a reduced phosphorylation levels of FAK, Src, and the downstream kinases AKT, MEK1/2 and p38-MAPK, as well as in the matrix metalloproteinases MMP2 and MMP9. This mechanism of action differs from that of TMZ, which at equipotent concentrations causes a non-specific blockade in the protein synthesis [2]. These studies suggest that the mechanisms of action of both drugs are different, and that NEO212 acts as a multi-target drug, potentially resulting in improved anti-tumor efficacy and lower risk of drug resistance and tumor recurrence. FAK/Src route regulates the EMT process, a prerequisite for cell migration, invasion, and metastasis. Gene expression analysis of epithelial and mesenchymal markers suggest that NEO212 reverts the epithelial-to-mesenchymal transition (EMT) process, mainly by upregulating several genes commonly downregulated during EMT [2].

*In vivo* efficacy studies have shown that NEO212 decreases tumor progression by reducing invasion of GSC and thus prolonging survival time of mice, to a greater extent than TMZ. *In vivo* toxicity studies of NEO212 determined the maximum tolerated dose, demonstrating that the experimental doses were not toxic to the mice. Pharmacokinetic studies from tumor-bearing and normal brains, in conjunction with the *in vivo* efficacy data, confirmed that NEO212 is delivered to the brain following subcutaneous administration, indicating that NEO212 crosses the blood-brain-barrier. NEO212 was retained in tumor-bearing brains after 120 minutes while it was washed out in normal brains, suggesting a possible binding or accumulation of NEO212 in tumor cells compared to normal brain tissue. *Ex vivo* immunostaining of the brains showed clear borders between the tumor and normal brain parenchyma in NEO212-treated mice, while TMZ-treated mice exhibited tumor cells invading the normal brain. These results demonstrated NEO212 effectiveness in reducing migration and invasion in a preclinical setting.

Clinically, we foresee that NEO212 would be active at lower concentrations compared to TMZ, while exhibiting minimal toxicity. Its ability to block GSC migration and invasion *in vivo* [2], as well as its selective cytotoxicity for tumor cells [5] are unusual in chemotherapy, making NEO212 an ideal candidate for the treatment of newly diagnosed and TMZ-resistant recurrent gliomas. As NEO212 cytotoxicity is independent of the status of the DNA repair protein MGMT, the main mechanism conferring resistance to TMZ [7], it could be applied to all patients with malignant gliomas. Our recent discoveries have implications in terms of how NEO212 would be administered in clinical settings. If NEO212 is administered at a similar schedule to the Stupp protocol used for TMZ (5 days on, 23 days off), higher doses of NEO212 could be administered for cytotoxicity for 5 days, followed by cycles of 23 days with lower doses for anti-migration/invasion properties.
